# Topological Signal Processing from Stereo Visual SLAM

**DOI:** 10.3390/s25196103

**Published:** 2025-10-03

**Authors:** Eleonora Di Salvo, Tommaso Latino, Maria Sanzone, Alessia Trozzo, Stefania Colonnese

**Affiliations:** Department of Information Engineering, Electronics and Telecommunications, Sapienza University of Rome, 00184 Rome, Italy; eleonora.disalvo@uniroma1.it (E.D.S.);

**Keywords:** Graph Signal Processing (GSP), Harmonic functions, stereo camera, Topological Signal Processing (TSP), Visual Simultaneous Localization and Mapping (V-SLAM)

## Abstract

Topological signal processing is emerging alongside Graph Signal Processing (GSP) in various applications, incorporating higher-order connectivity structures—such as faces—in addition to nodes and edges, for enriched connectivity modeling. Rich point clouds acquired by multi-camera systems in Visual Simultaneous Localization and Mapping (V-SLAM) are typically processed using graph-based methods. In this work, we introduce a topological signal processing (TSP) framework that integrates texture information extracted from V-SLAM; we refer to this framework as TSP-SLAM. We show how TSP-SLAM enables the extension of graph-based point cloud processing to more advanced topological signal processing techniques. We demonstrate, on real stereo data, that TSP-SLAM enables a richer point cloud representation by associating signals not only with vertices but also with edges and faces of the mesh computed from the point cloud. Numerical results show that TSP-SLAM supports the design of topological filtering algorithms by exploiting the mapping between the 3D mesh faces, edges and vertices and their 2D image projections. These findings confirm the potential of TSP-SLAM for topological signal processing of point cloud data acquired in challenging V-SLAM environments.

## 1. Introduction

Topological signal processing [1] is gaining momentum in different applications ranging from the characterization of brain networks [2] to the monitoring of physical systems [3,4]. Compared to GSP, TSP extends the representation by associating signals not only with nodes and edges, but also with higher-order connectivity structures such as faces, thereby enabling enriched connectivity modeling. Representing signals on topological domains remains an ongoing issue [5], particularly in the context of dictionary learning.

In this work, we propose a novel framework, termed TSP-SLAM, which establishes a direct link between the texture information provided by Visual Simultaneous Localization and Mapping (V-SLAM) and the topological signal processing of point clouds acquired through multi-camera systems. While V-SLAM has achieved significant advances for 3D reconstruction in static [6] and dynamic [7,8,9] scenarios, graph-based methods relying solely on node-to-node connectivity fail to capture higher-order structures such as planar surfaces and occlusions [10]. Higher-order topological representations, including faces and simplicial complexes, improve robustness and semantic consistency, and recent studies demonstrate that both clustering and geometric learning benefit from such enriched connectivity [10,11,12].

State-of-the-art extensions, such as the integration of deep learning [13] and hardware optimizations [14], further enhance robustness by adaptively handling visual features under varying conditions. SLAM outputs are increasingly enriched with value-aware geometric reconstructions, as in [15] where a dense neural point cloud model encodes attributes such as normals and intensity. Implicit neural surfaces [16] enable signal extraction on dense geometries, while Red Green and Blue-Depth (RGB-D) and Light Detection and Ranging (LiDAR) information in topology-based models [17] support loop closure and relocalization and enhance robustness and scalability. Graph-theoretic signatures (e.g., von Neumann entropy, spanning trees) [18] reduce uncertainty while lowering computational cost. With the growing adoption of real-time navigation and mapping for autonomous systems [19,20], V-SLAM enables on-the-fly mapping and safe adaptation to environmental changes [21] simultaneously performing localization and mapping using camera input [22,23] in dynamic autonomous system applications.

Within this context, the main contribution of this study is the introduction of TSP-SLAM, a texture-aware topological framework that extends the 2D-to-3D geometric mappings of V-SLAM to associate signals not only with individual points in the cloud, but also with higher-order topological structures. In addition, recent work such as [24] has demonstrated the use of topological descriptors for segmentation and recognition of objects from point clouds. While effective, these methods primarily rely on geometric information and do not exploit luminance or photometric texture cues. Our framework suggests that integrating luminance-based signals alongside geometric descriptors could further benefit tasks such as segmentation and recognition in point cloud processing. Specifically, TSP-SLAM supports the construction of signals over nodes, edges, and faces, thereby enabling advanced topological signal processing strategies. As a case study, we present Topological Multiscale Anisotropic Harmonic Filtering (T-MAHF), which extends to the topological domain a filtering technique (MAHF) originally introduced on graphs and applied successfully to denoising problems, thereby enhancing localization and mapping accuracy [25]. Numerical results confirm the effectiveness of the enriched representation achieved by TSP-SLAM for robust and expressive point cloud processing.

The remainder of this paper is organized as follows. Section 2 introduces the TSP-SLAM framework, providing a detailed description of the construction of topological structures from V-SLAM point clouds and the assignment of signals to nodes, edges, and faces. In Section 3, we present the mathematical formulation and implementation aspects of the T-MAHF algorithm. Section 4 reports the results of numerical experiments and comparative analyses, demonstrating the advantages of TSP-SLAM in terms of expressiveness of the resulting signal representations. Finally, Section 5 concludes the paper by summarizing the main contributions and outlining potential directions for future research.

## 2. Topological Signal Processing Framework from Visual SLAM

In this section, we introduce TSP-SLAM, a topological signal processing framework [1] enriched with texture information extracted from visual SLAM.

### 2.1. Signal Model in Visual-SLAM

**Figure 1:**
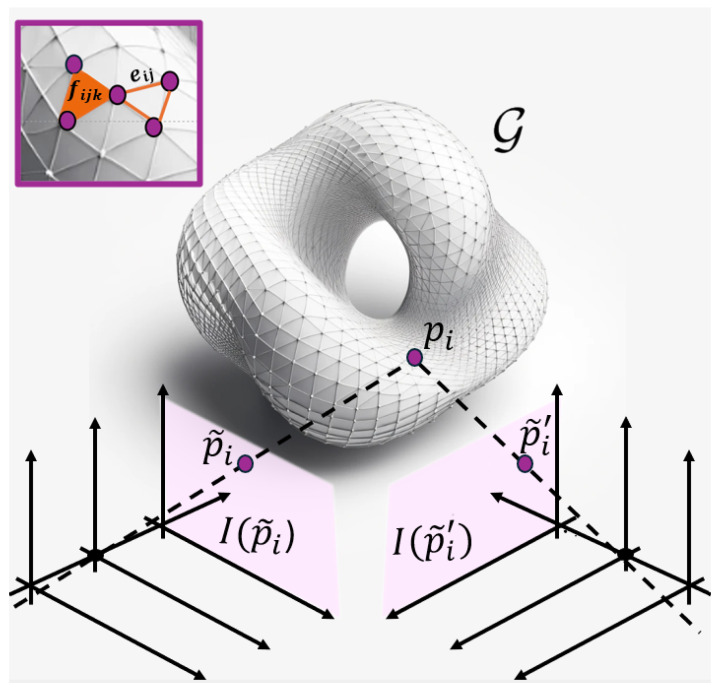
Illustration of the stereo acquisition architecture employed for 3D scene reconstruction from synchronized video streams: the figure depicts the left and right image planes capturing a 3D object, represented as a mesh, and the projections of a 3D point p onto both views, i.e., $\tilde{p}$, ${\tilde{p}}^{\prime }$.

Herein, we review the system outlined in Figure 1, reflecting a commonly adopted acquisition and tracking architecture as in [7]. The acquisition setup is stereo-based and relies on two sensors: a left camera, taken as reference, and a right camera, employed for 3D reconstruction. The left and right camera images are obtained by sampling the ideal bidimensional signals

(1) $$I\left(\tilde{p}\right),\;I^{\prime }\left({\tilde{p}}^{\prime }\right),\;\tilde{p},{\tilde{p}}^{\prime }\in R^{2}$$

on a discrete M×N grid. In V-SLAM, localization and mapping techniques are used to reconstruct a 3D point cloud from the stereo image disparity map, and the point features are used for object tracking and classification purposes. After stereo acquisition using the two calibrated cameras (left and right), which capture synchronized images, a set of distinctive keypoints is extracted from both images using feature extraction techniques such as ORB, SIFT, or BRISK. Let us denote these 2D points as ${\tilde{p}}_{i}$ for the left image and ${{\tilde{p}}_{i}}^{\prime }$ for the right image, with i=0,…,N−1, where *i* indexes the detected feature points in the left and right images. Feature matching algorithm, implemented through Hamming distance, is used to associate points between the two images, allowing the computation of the disparity for each match as $d_{i}=\left|\right|{\tilde{p}}_{i}-\;{\tilde{p}}_{i}^{\prime }{\left|\right|}_{1}$. In homogeneous coordinates—where affine transformations can be represented as matrix multiplications—each point cloud vertex is represented by augmenting its 3D coordinates with a unitary component. The pin-hole model describes the relationship between each three-dimensional point $p_{i}\in R^{3},\;i=0,\ldots N-1$ and its projection onto the images, depending on the cameras’ intrinsic parameters such as optical center, focal length, and optical axes. For a point cloud vertex at depth *z* from the camera axis origin, taking into account the camera focal length *l*, the relation between the 3D coordinates $p_{i}={\left[p_{ix},p_{iy},p_{iz}\right]}^{T}$ and their 2D counterparts ${\tilde{p}}_{i}={\left[u_{i},v_{i}\right]}^{T}$ in the image domain are written as follows:

(2) $$p_{iz}\left[\begin{array}{c} {\tilde{p}}_{i}\\ 1 \end{array}\right]=\underset{P}{\underset{︸}{\left[\begin{array}{cccc} \frac{l}{s_{x}} & \frac{l}{s_{x}\cot\left(\theta \right)} & u_{0} & 0\\ 0 & \frac{l}{s_{y}} & v_{0} & 0\\ 0 & 0 & 1 & 0 \end{array}\right]}}\left[\begin{array}{c} p_{i}\\ 1 \end{array}\right]$$

where the perspective projection matrix P accounts for the origin of the image coordinate system at the principal point ${\left[u_{0},v_{0}\right]}^{T}$, the pixel dimensions $\left(s_{x},s_{y}\right)$, and the angle between the axes $\theta \approx \frac{\pi }{2}$ arising from manufacturing imperfections. The outcome of this process is a 3D point cloud of vertices $p_{i}\in R^{3},\;i=0,\ldots N-1$ the vertices of the point cloud. A real-valued attribute signal is associated with each vertex, representing the color components of the points, or other attributes inherited from the original imaging system (e.g., temperature, reflectance, etc.).

In the following, we assume that the signal associated with the *i*-th point cloud vertex is the luminance of the corresponding 2D point as captured by the reference (left) camera. The point cloud can be further structured into a surface mesh using Delaunay triangulation, which ensures geometric consistency and avoids poorly shaped (e.g., degenerate or sliver) triangles. Based on these positions, we can introduce the topological signal processing framework as follows. The notation is summarized in Table 1.

### 2.2. Topological Signal Processing from V-SLAM Data

Let us define the graph G associated with the point cloud as G=(V,E), where V is the set of *N* point cloud vertices $p_{i}$, with i=1,…,N, and E is the set of edges. A topologically enriched graph structure is described by the vertex-to-edge incidence matrix $B_{1}\in R^{\left|V|\times |E\right|}$ and the edge-to-face incidence matrix $B_{2}\in R^{\left|E|\times |F\right|}$. Furthermore, the vertex Laplacian $L_{0}=B_{1}B_{1}^{⊤}$, and the edge Laplacian $L_{1}=B_{1}^{⊤}B_{1}+B_{2}B_{2}^{⊤}$ act as discrete differential operators on signals defined on the topological space.

**Figure 2:**
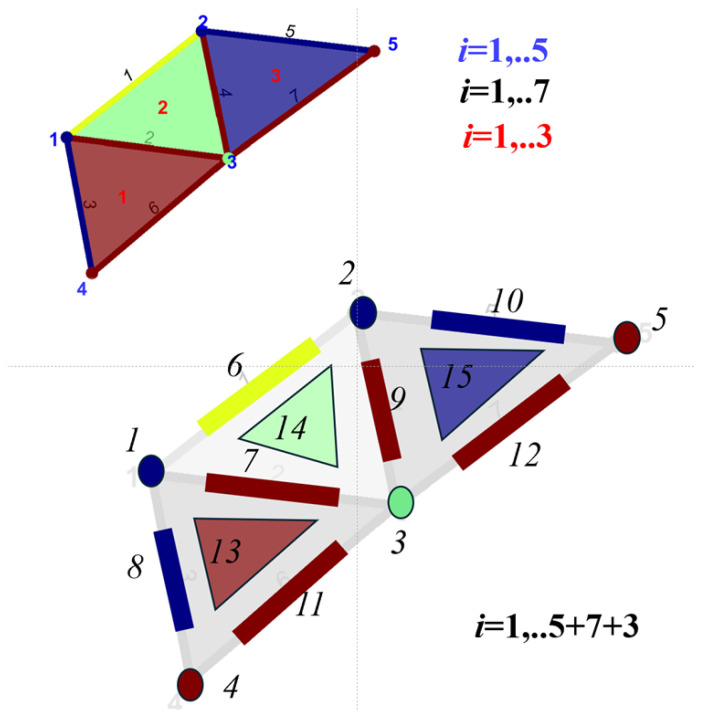
Graph G, with N=5 nodes, $N_{e}\;=\;7$ edges, and $N_{f}\;=\;7$ faces: $N+N_{e}+N_{f}=15$ signal values are defined on the node, edge, and face elements. Topological signal processing operates on these signals by exploiting neighborhood relationships established in the graph.

As a toy example, we illustrate these concepts in Figure 2, which represents a graph G with N=5 nodes, $N_{e}\;=\;7$ edges, and $N_{f}\;=\;7$ faces. The $N_{e}\times N$ vertex-to-edge incidence matrix $B_{1}$ and the $N_{f}\times N_{e}$ edge-to-face incidence matrix $B_{2}$ are defined as follows:

(3) $$B_{1}=\left[\begin{array}{ccccccc} 1 & 1 & 1 & 0 & 0 & 0 & 0\\ 1 & 0 & 0 & 1 & 1 & 0 & 0\\ 0 & 1 & 0 & 1 & 0 & 1 & 1\\ 0 & 0 & 1 & 0 & 0 & 1 & 0\\ 0 & 0 & 0 & 0 & 1 & 0 & 1 \end{array}\right],\;B_{2}=\left[\begin{array}{ccc} 0 & 1 & 0\\ 1 & 1 & 0\\ 1 & 0 & 0\\ 0 & 1 & 1\\ 0 & 0 & 1\\ 1 & 0 & 0\\ 0 & 0 & 1 \end{array}\right].$$

In topological signal processing, signals are assigned to different topological elements of the graph, i.e., nodes, edges, and faces, enabling topological signal processing tasks like smoothing or denoising in the edge and face domains. For instance, with reference to the toy example in Figure 2, overall, $N+N_{e}+N_{f}=15$ signal values can be defined on the node, edge, and face elements.

Let us denote by $s(p_{i})$ the signal at *i*-th node, by $s(e_{j})$ the signal at *j*-th graph edge, and by $s(f_{k})$ the signal at *k*-th graph face.

In general, at the node level, signals $s(p_{i})$ may represent sensor data directly acquired at each point vertex, e.g., RGB color values. At the edge level, signals $s(e_{j})$ may encode pairwise relationships between adjacent vertices, e.g., geometric descriptors such as their Euclidean distance or the differences in vertex attributes. At the face level, signals $s(f_{k})$ can capture higher-order geometric features, such as surface normals or texture patterns extracted from the original image.

In TSP-SLAM, we propose that the signals at different levels are obtained from the texture side information available through the V-SLAM framework. In detail, the signal at the nodes $p_{i}$, edges $e_{j}$ and faces $f_{k}$ are derived from the photometric values pertaining to their projections, i.e., the points ${\tilde{p}}_{i}$, segments ${\tilde{e}}_{j}$ or triangles ${\tilde{f}}_{k}$ lying in the original reference image used by the V-SLAM technique.

The way values on edges $e_{i}$ and faces $f_{k}$ are determined from photometric information associated with their projected segments, ${\tilde{e}}_{j}$ and ${\tilde{f}}_{k}$, is not unique. Each topological element corresponds to a set of pixels in the 2D domain: the *j*-th edge $e_{j}$ corresponds to the set $E_{j}$ of pixels along the segment ${\tilde{e}}_{j}$, while the *k*-th face $f_{k}$ corresponds to the set $F_{k}$ of pixels within the triangle ${\tilde{f}}_{k}$. A reasonable approach is to associate each topological element $e_{i}$ and face $f_{k}$ with a signal obtained by averaging the photometric values of the corresponding set of pixels $E_{j}$, $F_{k}$ in the image plane. Without loss of generality, we adopt this method in the following, where we show how TSP-SLAM enables topological signal processing on V-SLAM-acquired point clouds, assigning distinct signal information to vertices, edges, and faces. Nevertheless, alternative strategies for defining vector-valued signals [1] on topological elements can be designed, and their exploration is left for future work.

In the following, without loss of generality, to define the signal related to edge and faces, we exploit the information available from the TSP-SLAM framework. Specifically, we define the signal $s(p_{j})$ at the *j*-th vertex as the signal of its 2D projection

(4) $$s\left(p_{j}\right)=s\left({\tilde{p}}_{j}\right),$$

the signal $s(e_{j})$ at the *j*-th edge as the average of the signal over the set $E_{j}$ of pixels belonging to the segment ${\tilde{e}}_{j}$,

(5) $$s\left(e_{j}\right)=\frac{1}{|E_{j}|}\sum_{\tilde{p}\in E_{j}}s\left({\tilde{p}}_{j}\right),$$

the signal $s(f_{j})$ at the *k*-th face $f_{k}$ as the average on the set $F_{j}$ of pixels within the triangle ${\tilde{f}}_{j}$

(6) $$s\left(f_{j}\right)=\frac{1}{|F_{j}|}\sum_{\tilde{p}\in F_{j}}s\left({\tilde{p}}_{j}\right)$$

## 3. Topological Harmonic Filtering in TSP-SLAM

Here, we show an application of TSP-SLAM to extend anisotropic graph filtering to topological spaces. Specifically, we extend a filtering technique previously introduced for images and signals on graphs to the topological space, and we show how it can leverage the side information provided by TSP-SLAM. Thanks to the information acquired by TSP-SLAM, each topological level—vertex, edge, face—carries its own type of signal, allowing for a rich and flexible representation of the geometry and semantics of the point cloud.

Multiscale harmonic filters were formerly introduced and widely applied in image processing. Among these, Complex Harmonic Filters [26] operate over a 2D spatial domain as a function of radial distance and angular orientation. The filtering is written as follows:

(7) $$I_{CHF}\left(\tilde{p}\right)=\sum_{n=0}^{N-1}{\tilde{h}}^{\left(m\right)}\left(\tilde{p},{\tilde{p}}_{n}\right)I\left({\tilde{p}}_{n}\right)\;$$

where ${\tilde{h}}^{\left(m\right)}\left(\tilde{p},{\tilde{p}}_{n}\right)$ is defined as a separable function:

(8) $${\tilde{h}}^{\left(m\right)}\left(\tilde{p},{\tilde{p}}_{n}\right)=g_{m}\left(|\right|\tilde{p}-{\tilde{p}}_{n}\left||\right)e^{j\;m\arg\{\tilde{p}-{\tilde{p}}_{n}\}}$$

$g_{m}\left(\cdot \right)$ is a radial envelope—typically an isotropic Gaussian kernel—ensuring spatial localization, and the exponential term encodes angular selectivity. For m=0, the filter is purely radial and yields a real-valued low-pass response, effectively acting as a smoothing operator, and for m=1, the angular term introduces directional sensitivity, allowing the filter to highlight edge-like structures oriented along specific directions.

Recently, the harmonic filtering approach has been extended to non-Euclidean domain in [27], where multiscale anisotropic filters have been introduced. The Multiscale Anisotropic Harmonic Filters (MAHF) of *m*-th order and centered at $p_{i}$ act on a point cloud signal $s(p_{i})$ as follows:

(9) $$r\left(p_{i}\right)=\sum_{i=0}^{N-1}h^{\left(m\right)}\left(p_{i},p_{j}\right)\;s\left(p_{j}\right)$$

and it is defined as a function of the geodesic distance metric between $p_{i}$ and $p_{j}$, and of the angular coordinate of the *j*-th vertex on the graph G tangent plane centered at $p_{i}$ in the following formulas:

(10) $$h^{\left(m\right)}\left(p_{i},p_{j}\right)=K_{t}^{\left(G\right)}\left(p_{i},p_{j}\right)\left(\cos\left(m\;\varphi^{\left(G\right)}\left(p_{i},p_{j}\right)\right)+j\;\sin\left(m\;\varphi^{\left(G\right)}\left(p_{i},p_{j}\right)\right)\right)\;$$

where $\varphi^{\left(G\right)}\left(p_{i},p_{j}\right)$ is the angular coordinate of $p_{j}$ on the tangent plane in $p_{i}$. The function $K_{t}^{\left(G\right)}\left(p_{i},p_{j}\right)$ is a real weighting function depending on the connectivity of the point cloud graph *G*. Specifically, $K_{t}^{\left(G\right)}\left(p_{i},p_{j}\right)$ is the so-called heat diffusion kernel, formulated based on the theory of heat diffusion over smooth surfaces, and it is defined as follows. Let $U=[u_{0},\ldots u_{N-1}]$ denote the eigenvectors of the graph Laplacian $L_{0}$. The kernel is computed as

(11) $$K_{t}^{\left(G\right)}\left(p_{i},p_{j}\right)=\sum_{n=0}^{N-1}e^{-\lambda_{n}t}u_{n}\left[i\right]u_{n}\left[j\right].$$

Hence, $K_{t}^{\left(G\right)}\left(p_{i},p_{j}\right)$ is related to the differences between the *i*-th and *j*-th coefficients across the eigenvectors of the Laplacian $L_{0}$, and it is larger when the points $p_{i},p_{j}$ belong to strongly connected areas [25]. MAHF has been applied to signals on point clouds for different tasks, such as point cloud denoising [25] or visual quality evaluation [28]. Still, MAHF filters rely on accurate estimation of the Laplacian associated with the point cloud, in turn depending on the quality of the triangulation algorithm. Point clouds from V-SLAM techniques often capture different objects at a wide set of different distances. This hinders the development of a regular mesh model by conventional triangulation techniques. Hence, useful feature extractors such as MAHF, which proved useful in application like denoising or point cloud quality evaluation, can fail to extract features due to sensitivity to Laplacian/mesh errors.

Making use of the side information provided by signals on edges and faces can lead to a richer estimate of the point cloud features. Specifically, we leverage the side information provided in TSP-SLAM to build an enhanced version of MAHF, suited to topological signal processing, which we refer to as T-MAHF.

In T-MAHF, we leverage the TSP-SLAM framework to generalize MAHF by exploiting side information available at the graph G associated with the point cloud, as detailed below. We define the output of the T-MAHF topological filter at the *i*-th vertex as follows:

(12) $$r\left(p_{i}\right)=\underset{\mathrm{signal}\;\mathrm{on}\;\mathrm{vertices}}{\underset{︸}{\sum_{j=0}^{N_{f}-1}h^{\left(m\right)}\left(p_{i},p_{j}\right)\;s\left(p_{j}\right)}}+\underset{\mathrm{signal}\;\mathrm{on}\;\mathrm{edges}}{\underset{︸}{\sum_{j=0}^{N_{f}-1}h^{\left(m\right)}\left(p_{i},e_{j}\right)\;s\left(e_{j}\right)}}+\underset{\mathrm{signal}\;\mathrm{on}\;\mathrm{faces}}{\underset{︸}{\sum_{j=0}^{N_{f}-1}\left(p_{i},f_{j}\right)\;s\left(f_{j}\right)}}$$

where, for a generic topological element $x_{j}$ representing a node, an edge, or a face, the function $h^{\left(m\right)}\left(p_{i},x_{j}\right)$ is written as follows:

(13) $$h^{\left(m\right)}\left(p_{i},x_{j}\right)=K_{\tau }^{\left(T\right)}\left(p_{i},x_{j}\right)\left(\cos\left(m\;\varphi^{\left(T\right)}\left(p_{i},x_{j}\right)\right)+j\;\sin\left(m\;\varphi^{\left(T\right)}\left(p_{i},x_{j}\right)\right)\right)$$

where $K_{\alpha }^{\left(T\right)}\left(p_{i},x_{j}\right)$ a real weighting function depending on a non-Euclidean distance metric between $p_{i}$ and a neighboring element $x_{j}$, i.e., on the connectivity of the topological space T associated with the point cloud, and $\varphi^{\left(T\right)}\left(p_{i},x_{j}\right)$ a measure of angular distance in the tangent plane at the *i*-th vertex.

The TSP-SLAM framework allows us to compute the functions $K_{\alpha }^{\left(T\right)}\left(p_{i},x_{j}\right)$ and $\varphi^{\left(T\right)}\left(p_{i},x_{j}\right)$ appearing in $h^{\left(m\right)}\left(p_{i},x_{j}\right)$ with approximate values computed in the TSP-SLAM framework. To this end, let us develop the neighborhood system of the *i*-th vertex in the topological space, as established by the incidence matrices $B_{1}$ and $B_{2}$. Let us denote the neighborhood of the *i*-th vertex by introducing the set $\eta_{i}^{\left(f\right)}$ of the neighboring faces, i.e., faces that are incident on the vertex:

(14) $$\eta_{i}^{\left(f\right)}=\left\{f_{k}\;|\;{\left[B_{2}B_{1}\right]}_{k,i}\neq 0\;\mathrm{for}\;\mathrm{at}\;\mathrm{least}\;\mathrm{one}\;k\right\}.$$

Then, we introduce the set $\eta_{i}^{\left(e\right)}$ of neighboring edges as those belonging to the incident faces in $\eta_{i}^{\left(f\right)}$

(15) $$\eta_{i}^{\left(e\right)}=\left\{e_{j}\;|\;B_{2}\left(j,k\right)\neq 0\;\mathrm{for}\;\mathrm{any}\;f_{k}\in \eta_{i}^{\left(f\right)}\right\}.$$

Finally we denote the set $\eta_{i}^{\left(p\right)}$ of neighboring nodes:

(16) $$\eta_{i}^{\left(p\right)}=\left\{p_{j}\;|\;B_{1}\left(i,k\right)\cdot B_{1}\left(j,k\right)\neq 0\;\mathrm{for}\;\mathrm{at}\;\mathrm{least}\;\mathrm{one}\;k\right\}.$$

In the following experiments, we set the weighting function as follows:

(17) $$K_{\alpha }^{\left(T\right)}\left(p_{i},x_{j}\right)=\left\{\begin{array}{cc} 1 & x_{j}\in \eta_{i}^{\left(\cdot \right)}\\ 0 & \mathrm{otherwise} \end{array}\right.$$

i.e., $K_{\alpha }^{\left(T\right)}\left(p_{i},x_{j}\right)$ is an indicator function that specifies whether $x_{j}$—which can be a node, face or edge—belongs to a neighborhood system of the *i*-th vertex or not. It should be noted that with this definition, the values of the weighting function in Equation (17) are larger (equal to one) for one-hop connected topological elements, and zero otherwise. This choice provides a hard approximation of topology element interactions extending the soft definition in (11). More generally, one could define the weighting function as a decreasing function of the topological distance $d_{T}\left(p_{i},x_{j}\right)$, namely

(18) $$K_{\alpha }^{\left(T\right)}\left(p_{i},x_{j}\right)=g\left(d_{T}\left(p_{i},x_{j}\right)\right),\;g:R^{+}\to \left[0,1\right],\;g\left(0\right)=1,\;g\left(d\right)↓0\;\;\mathrm{as}\;d\to \infty ,$$

where the indicator function adopted in this work corresponds to the simplest binary instance of this general formulation.

The T-MAHF expression is then rewritten as follows:

(19) $$r\left(p_{i}\right)=\underset{N\;\mathrm{vertices}}{\underset{︸}{\sum_{j\in \eta_{i}^{\left(p\right)}}h^{\left(m\right)}\left(p_{i},p_{j}\right)\;s\left(p_{j}\right)}}+\underset{N_{e}\;\mathrm{edges}}{\underset{︸}{\sum_{j\in \eta_{i}^{\left(e\right)}}h^{\left(m\right)}\left(p_{i},e_{j}\right)\;s\left(e_{j}\right)}}+\underset{N_{f}\;\mathrm{faces}}{\underset{︸}{\sum_{j\in \eta_{i}^{\left(f\right)}}h^{\left(m\right)}\left(p_{i},f_{j}\right)\;s\left(f_{j}\right)}}$$

Let us observe that $r\left(p_{i}\right)=M_{i}\;e^{j\vartheta_{i}}$ is a complex number, whose magnitude describes the intensity of the local variation and whose phase is related to the direction of the variation. In addition, we approximate the angular distance metrics $\varphi^{\left(T\right)}\left(p_{i},p_{j}\right)$, $\varphi^{\left(T\right)}\left(p_{i},e_{j}\right)$, and $\varphi^{\left(T\right)}\left(p_{i},f_{j}\right)$ with their 2D counterparts. Specifically, we introduce the following approximations:

(20) $$\varphi^{\left(T\right)}\left(p_{i},p_{j}\right)\approx \psi \left({\tilde{p}}_{i},{\tilde{p}}_{j}\right),$$

(21) $$\varphi^{\left(T\right)}\left(p_{i},e_{j}\right)\approx \psi \left({\tilde{p}}_{i},\beta_{j}^{\left(e\right)}\right),\;\beta_{j}^{\left(e\right)}=\frac{1}{|E_{j}|}\sum_{\tilde{p}\in E_{j}}{\tilde{p}}_{j}$$

and

(22) $$\varphi^{\left(T\right)}\left(p_{i},f_{j}\right)\approx \psi \left({\tilde{p}}_{i},\beta_{j}^{\left(f\right)}\right),\;\beta_{j}^{\left(f\right)}=\frac{1}{|F_{j}|}\sum_{\tilde{p}\in F_{j}}{\tilde{p}}_{j}$$

where $\beta_{j}^{\left(e\right)}$ and $\beta_{j}^{\left(f\right)}$ are the barycenters of the edge and faces in the neighborhood systems of the vertex *i*. The above quantities are directly available in the 2D domain, and in the following we show the limits within which the above approximation stands.

As for the computational architecture, the proposed MAHF and T-MAHF methods share an initial V-SLAM feature detection and sparse reconstruction, producing 3D points of the scene. MAHF operates on the reconstructed mesh, computing the Laplacian and its eigenvectors to build the heat kernel, which is combined with 3D angular information from mesh normals to produce a complex-valued filtered signal and enable 3D angle computation. This fully exploits the 3D structure but has higher computational cost. T-MAHF, instead, although relying on the mesh topology, works on the 2D image plane, performing 2D angular filtering without spectral decomposition. While computationally lighter, it captures less geometric information, as 3D surface variations are not explicitly modeled.

To sum up, TSP-SLAM allows building a texture dictionary suited to topological point cloud signal processing. The topology-related dictionary is built by extending techniques used in simultaneous localization and mapping algorithms, and adapting it to the point cloud topology description. This is useful for several developments, where non-Euclidean operators can be applied for processing purposes, including topological neural network architecture as discussed in [29].

## 4. Numerical Results

**Figure 3:**
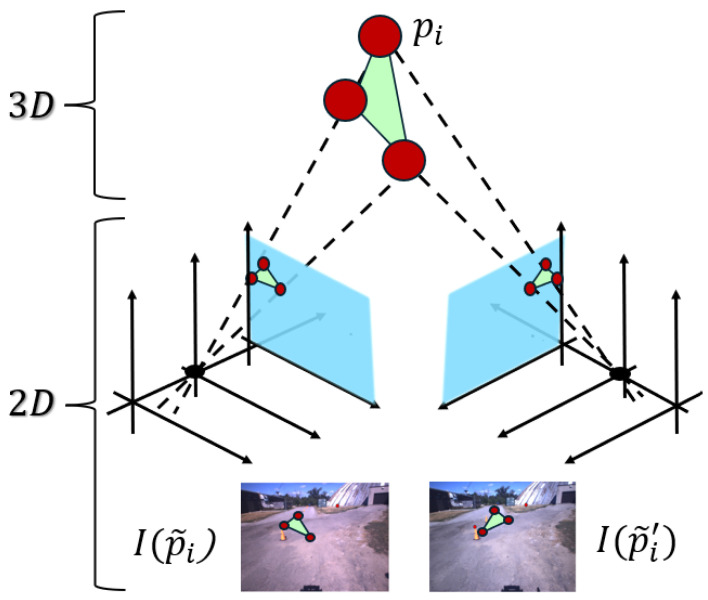
Schematic illustration of the recovery of 3D topological structure from 2D measurements: Starting from 2D observations of projected point triplets in a stereo image pair (bottom row), one can recover information about the underlying 3D triangular structure (top center). The middle row shows the schematic projection planes, where the corresponding left and right image coordinates, respectively ${\tilde{p}}_{i}\;=\;{\left[u_{i},v_{i}\right]}^{T}$ and ${\tilde{p}}_{i}^{\prime }\;=\;{\left[u_{i}^{\prime },v_{i}^{\prime }\right]}^{T}$, are detected. By leveraging geometric constraints and correspondences between the two views, it becomes possible to infer the spatial configuration of the original 3D surface, enabling the reconstruction of its topological structure.

Herein, we provide a set of results describing how the TSP-SLAM framework is built on real data. We then show how it can be used for topological processing by presenting the application of T-MAHF, with reference to the dataset in [30]. Figure 3 illustrates the topology acquisition from measurements. Starting from 2D observations of projected point triplets in a stereo pair of images (bottom row), one can recover information about the underlying 3D triangle structure (top center). The middle row shows the schematic projection planes, where the corresponding left and right image coordinates, respectively $\left[u_{i},v_{i}\right]$ and $\left[u_{i}^{\prime },v_{i}^{\prime }\right]$, are detected.

The two images in [30] used for topological processing were acquired with a stereo camera configuration, featuring a 0.24 m baseline and a resolution of 512×384 pixels. For keypoint detection and correspondence estimation, we adopted the method in [7], with the following settings: (i) the focal length of the cameras was 387.77 pixels for both x- and y- axes; (ii) the principal point of the cameras was located at coordinates (257.446, 197.718), specified in pixels; (iii) the maximum horizontal displacement between keypoints was limited to 48 pixels in order to filter matches that could lead to excessively shallow depth once triangulation is performed. By leveraging geometric constraints and correspondences between the two views, we inferred the spatial position of the points on the original 3D surface as in [7].

**Figure 4:**
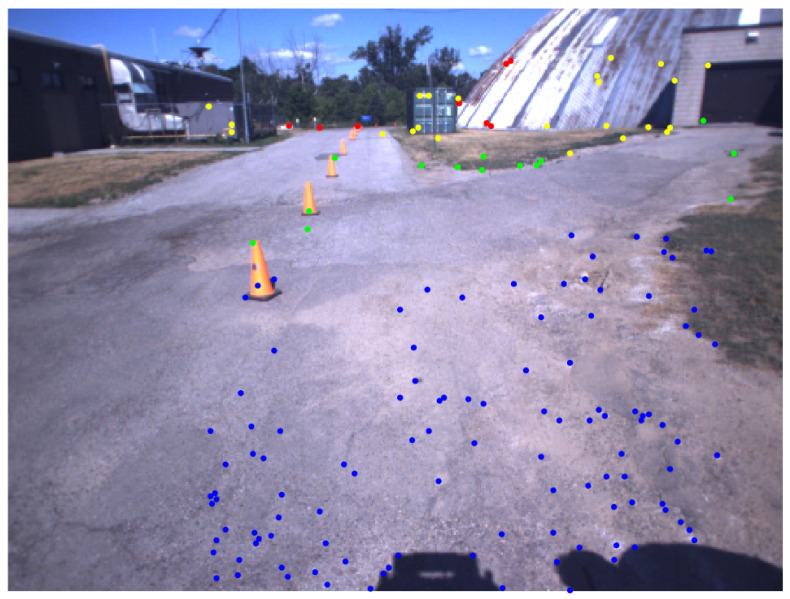
Detected keypoints from the stereo image pair: The features are extracted using ORB and are uniformly distributed across the image to ensure consistent spatial coverage. Each point is color-coded according to its depth value $p_{iz}$, namely, blue for $p_{iz}<8$, green for $8\leq p_{iz}<30$, yellow for $30\leq p_{iz}<60$, and red for $p_{iz}\geq 60$. This visualization highlights the spatial structure of the scene and reflects the depth-aware distribution of features, providing a meaningful basis for subsequent geometric and topological analyses.

Figure 4 represents the detected keypoints from the stereo image pair, with a pseudo-color indicating the distance of the corresponding 3D points with respect to the camera.

**Figure 5:**
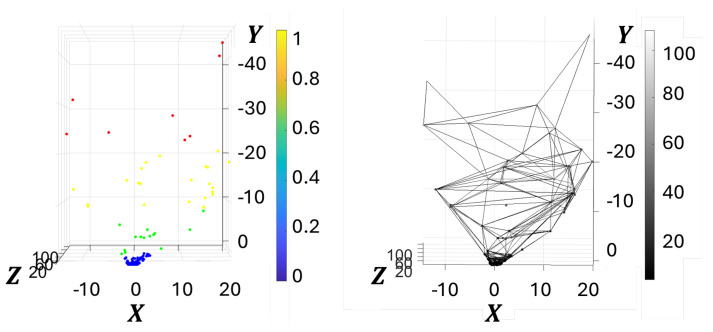
Three-dimensional point cloud and its corresponding topological structure: (**left**) The 3D point cloud $p_{i}$, i=0,…N−1, built on keypoints detected in the stereo image pair and triangulated into 3D coordinates; (**right**) associated topological structure. On the left, the point cloud is color-coded based on depth ($p_{iz}$, i=0,…N−1) values: blue for closer points and red for farther ones. On the right, a topological graph is constructed by connecting the 3D keypoints based on Crust algorithm, forming edges that reflect the underlying geometric structure. This representation enables the analysis of both geometric and topological features from image-derived 3D data.

The cloud of estimated 3D points is shown in Figure 5 (left), where the point color corresponds to its 3D depth $p_{iz}$. The point cloud was then equipped with a mesh using the Crust algorithm in [31], leading to a graph G associated to the 3D point cloud, as shown in Figure 5 (right). The graph was defined with binary edge weights. The graph incidence matrices $B_{1}$ and $B_{2}$, with values in {−1,0,1}, represent the graph connectivity in terms of edges and faces, thereby enabling the definition of a structure suitable for topological signal processing.

**Figure 6:**
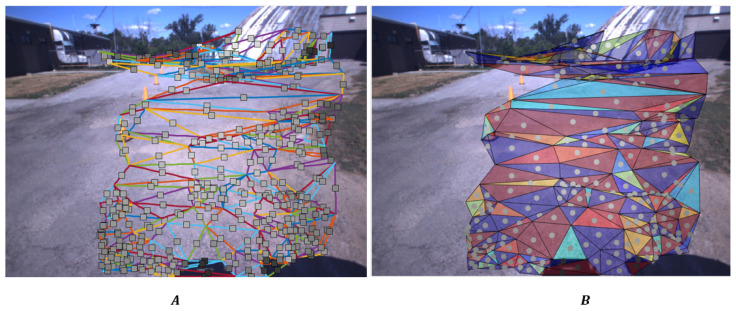
Illustration of the signals associated with edges and faces at their barycenters. In Panel (**A**), the signal $s(e_{j})$ associated with the edge $e_{j}$ is illustrated by a square located at the edge barycenter $\beta_{j}^{\left(e\right)}$, colored according to the average luminance over the edge pixels. In Panel (**B**), the signal $s(f_{j})$ associated with the face $f_{j}$ is shown at the barycenter $\beta_{j}^{\left(f\right)}$ of the triangle as a circle, colored according to the average luminance over the triangle pixels. The luminance levels of the squares and circles clearly reflect the brightness of the regions on which they fall.

In the TSP-SLAM framework, the topological elements (points, edges and faces) are naturally associated with the signals available in the 2D domain, as illustrated in Figure 6. Specifically, Figure 6A shows the projection of the 3D edges $e_{j},\;j=0,\ldots N_{e}-1$ in the 2D domain. At the barycenter $\beta_{j}$ of each edge, we plot a square indicating the average value of the luminance over the edge pixels. In the TSP-SLAM framework, this value will be used as the signal $s(e_{j})$ associated with the edge. Similarly, Figure 6B shows the projection of the 3D faces $f_{j},\;j=0,\ldots N_{f}-1$ in the 2D domain. At the barycenter $\beta_{j}$ a circle indicates the average value of the luminance over the face pixels, i.e., the signal $s(f_{j})$ that will be associated with the face.

With these positions, we present some results exemplifying possible applications of TSP-SLAM in T-MAHF filtering.

Firstly, it is worth observing that the heat diffusion function $K_{t}^{\left(G\right)}\left(p_{i},p_{j}\right)$, depending on a non-Euclidean distance metric between two 3D points $p_{i}$ and $p_{j}$, is correlated to the distance $d_{hop}\left(p_{i},p_{j}\right)$ between the two points in terms of graph hops.

**Figure 7:**
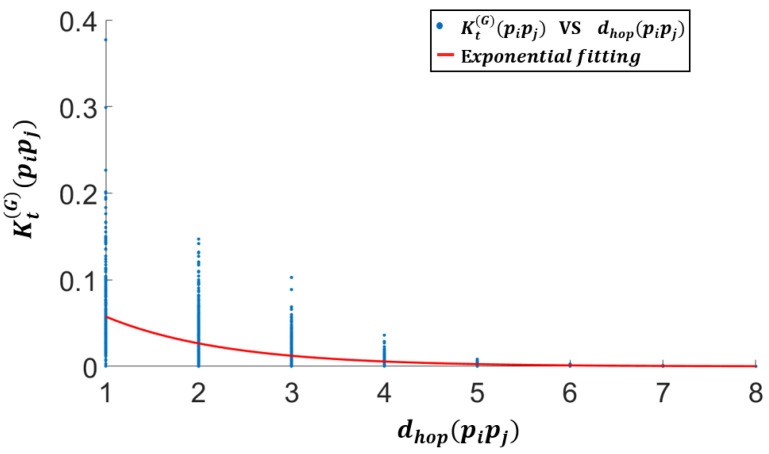
Heat kernel decay with respect to the distance between nodes: scatter plot of the values of the heat kernel $K_{t}^{\left(G\right)}\left(p_{i},p_{j}\right)$, versus the number of hops $\delta_{ij}$ between nodes $p_{i}$, $p_{j}$ (blue points), along with a first-order exponential approximation of the underlying relationship (red line).

This is illustrated in Figure 7 (blue circles), showing the scatter plot of the values of the heat diffusion kernel $K_{t}^{\left(G\right)}\left(p_{i},p_{j}\right)$—computed using a Chebyshev polynomial approximation of heat diffusion over the graph—versus the number of hops $d_{hop}\left(p_{i},p_{j}\right)$ between the same nodes. Figure 7 also shows a first-order exponential approximation of the underlying relationship. We observe a trend of exponential decay of the heat diffusion kernel $K_{t}^{\left(G\right)}\left(p_{i},p_{j}\right)$ versus the hop distances on the graph. This suggests that a reasonable weighting function can be obtained by retaining the closest topological elements.

This is the rationale behind the choice of approximating the T-MAHF weighting function with a neighborhood indicator function, as in (17).

**Figure 8:**
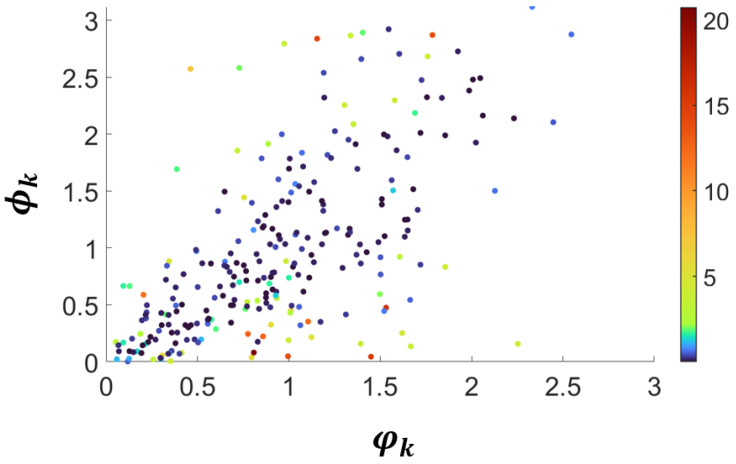
Scatter plot of the angles $\varphi (p_{i},p_{j})$ of the 3D faces versus the corresponding angles $\psi ({\tilde{p}}_{i},{\tilde{p}}_{j})$ computed in the 2D image plane: the point color represents the standard deviation of the depth ($\sigma_{z}$) measured over the coordinates of the 3D triangles. A low standard deviation indicates that the vertices approximately lie at the same depth, i.e., the 3D triangle is affine to the 2D triangle obtained via ray tracing.

Secondly, Figure 8 shows the scatter plot of the angles $\varphi^{\left(T\right)}\left(p_{i},p_{j}\right)$ measured in the 3D domain versus the corresponding angles $\psi ({\tilde{p}}_{i},{\tilde{p}}_{j})$ computed in the 2D image plane. Due to the ray tracing projection geometry underlying TSP-SLAM, the approximation is expected to hold when the angles belong to faces parallel to the image pane. As the faces are increasingly tilted in the 3D domain, the angles and their projection increasingly differ. This is evident in Figure 8, where the color of each point encodes the standard deviation of the depth ($p_{iz}$) coordinates of the triangle’s vertices in 3D space.

As expected, a low standard deviation indicates that the vertices lie (approximately) at the same depth, a condition in which the 3D triangle is approximately affine to the 2D projected triangle via ray tracing. For triangles with vertices at largely different depths, i.e., tilted with respect to the image plane, the 2D and 3D angles differ, but in most cases the scattered points lie along the bisectrix of the first quadrant, indicating that the 2D angles serve as an approximation of the corresponding 3D angles.

As illustrated in Figure 8, the discrepancy between 3D and 2D angular measurements increases with the depth variance of the face vertices, a phenomenon consistent with foreshortening effects reported in the structure-from-motion literature [32]. Reprojection errors and keypoint noise, as discussed in [33,34], are non-negligible in practical V-SLAM pipelines, but can be explicitly modeled to improve reconstruction reliability. Incorporating higher-order geometric primitives, such as faces and simplicial complexes, further helps to stabilize and refine point cloud representations by enforcing local planarity and structural consistency [35].

While exact 3D angle computation is computationally expensive, empirical evidence shows that 2D angular estimates align closely with their 3D counterparts when faces are approximately parallel to the image plane. Even for tilted faces, the scatter distribution remains concentrated around the bisector, indicating that 2D estimates provide a statistically reliable approximation. This trade-off allows TSP-SLAM to significantly reduce computational cost while maintaining geometric consistency at the topological level. Since the computation of the 3D angles $\varphi^{\left(T\right)}\left(p_{i},p_{j}\right)$ is computationally expensive, the fast approximation provided by the 2D estimated angles $\psi ({\tilde{p}}_{i},{\tilde{p}}_{j})$ can be adopted.

**Figure 9:**
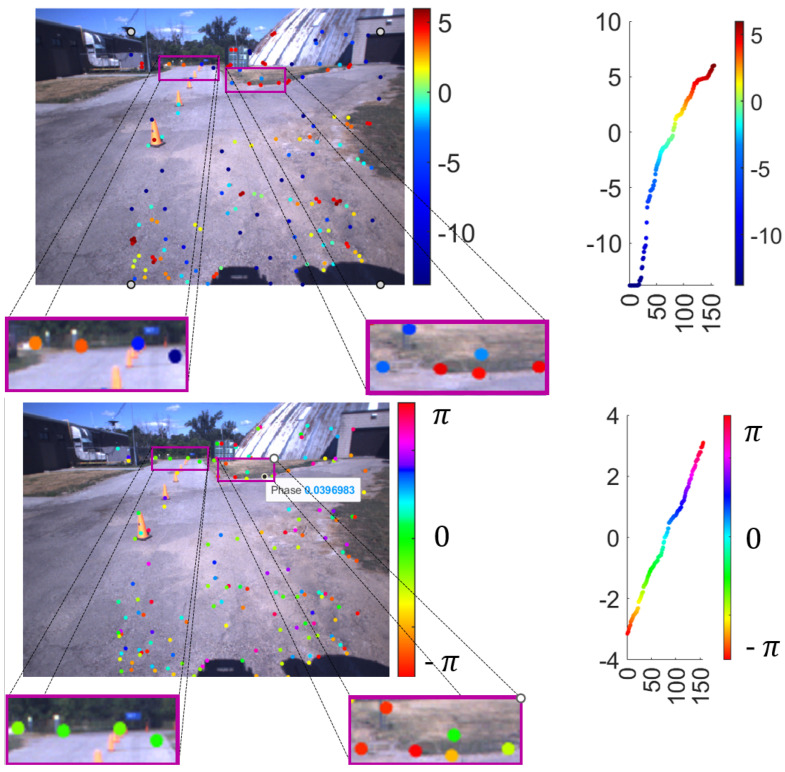
T-MAHF filter response: Absolute value (**top**) $M_{i}$, i=0,…N−1, and phase (**bottom**) $\vartheta_{i}$, i=0,…N−1, of the T-MAHF filter output, represented at the corresponding 2D keypoints ${\tilde{p}}_{i}$, i=0,…N−1. The values highlight local signal variations and their orientation, providing features for further processing (e.g., classification, recognition) in the 3D domain.

**Figure 10:**
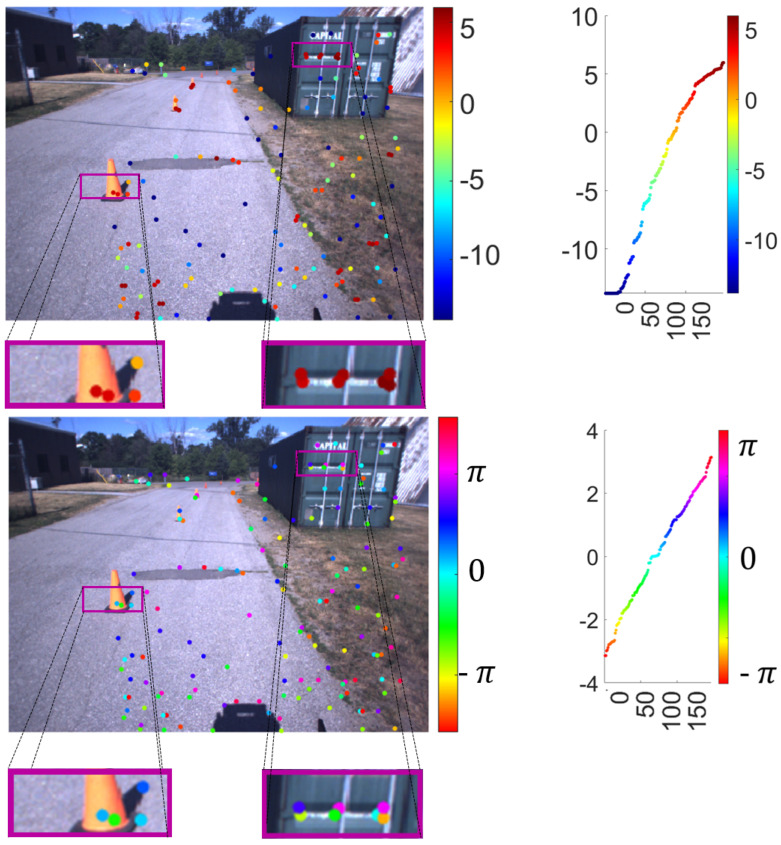
T-MAHF filter response on a different frame: The representation is the same as in previous Figure 8. Here, the analysis is performed on a different stereo frame with a different number of keypoints, demonstrating the algorithm’s performance under varying point cloud densities.

Finally, Figure 9 and Figure 10 present two examples of the result of topological harmonic filtering within the TSP-SLAM framework. For better visualization, the filter output at the 3D point ${\tilde{p}}_{i}$ is displayed at the corresponding keypoint ${\tilde{p}}_{i}$, overlaid on the image. The color of ${\tilde{p}}_{i}$ in the top panel represents the absolute value of the T-MAHF filter output on a logarithmic scale, i.e., $\log(M_{i})$, while the bottom panel represents, in the same manner, the phase of the T-MAHF filter output on a logarithmic scale, i.e., $\vartheta_{i}$. We observe that T-MAHF allows the ranking of points according to larger magnitudes $M_{i}$; for these points, the phase $\vartheta_{i}$ is an estimate of the direction of signal variation. In the bottom panel, detail in the right box, a few points characterized by larger magnitudes M−i are highlighted to illustrate the meaning of the phase component $\vartheta_{i}$. Although visually distinct, the selected points reveal a direction of the signal variation along angles of 0 (light green), π (red) and −π (fuchsia). Therefore, the variation corresponds to a horizontal discontinuity. The same applies to the detail in the left box, where horizontal or slightly tilted edges are recognized.

It is worth noting that, although visualized in the 2D domain for clarity, this information refers to the signal defined on the point cloud graph and its topology. Such information can be employed for various applications, including point cloud classification or loop closure recognition. At the same time, thanks to the TSP-SLAM framework, this information can be computed directly on 2D data, increasing robustness and reducing computational complexity compared to computations in the full 3D domain.

**Figure 11:**
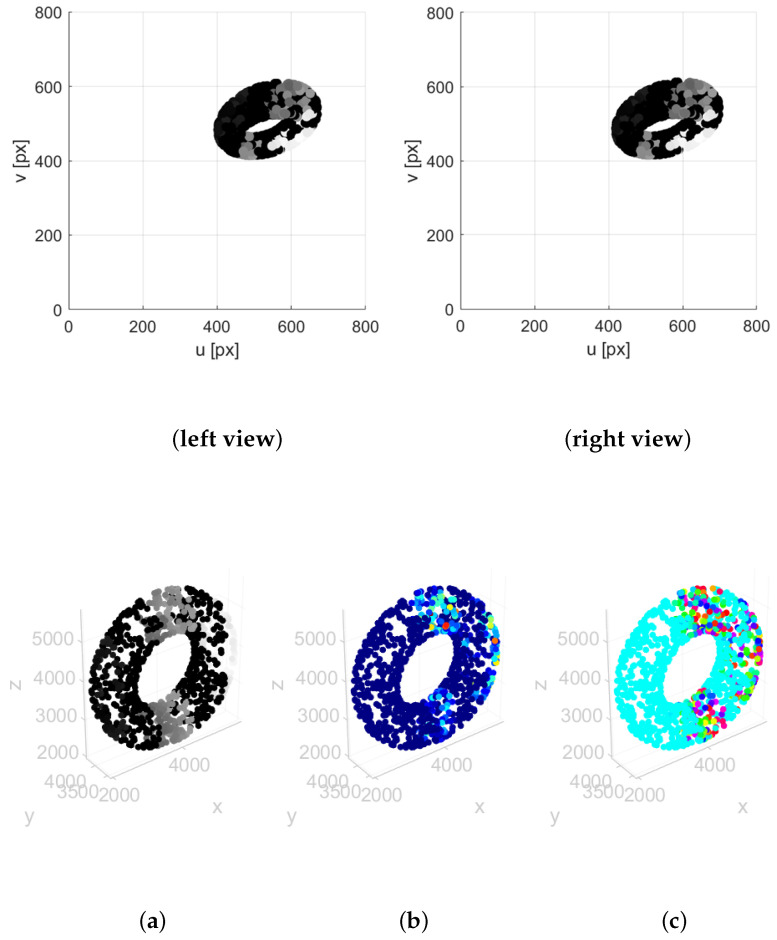
T-MAHF filter response on a toroidal point cloud: (**left view**,**right view**) Stereo projections of a synthetic toroidal grayscale point cloud with N=1000 points; (**a**–**c**) point cloud signal $s(p_{i})$, T-MAHF filter output $r(p_{i})$ magnitude $M_{i}$ and phase $\vartheta_{i}$.

**Figure 12:**
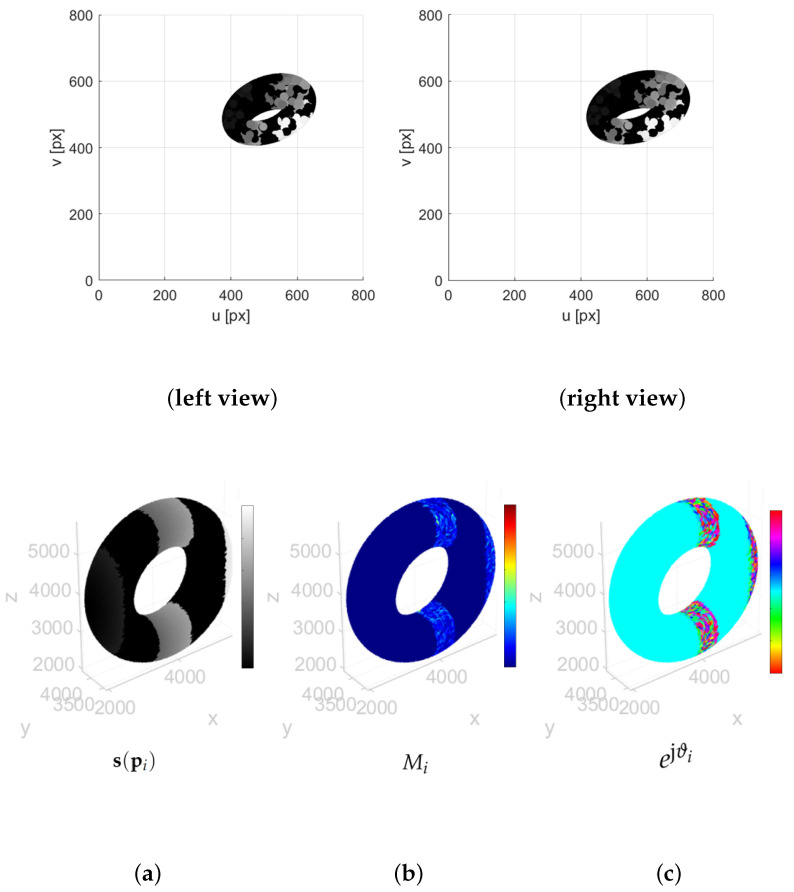
T-MAHF filter response on a toroidal point cloud: (**left view**,**right view**) stereo projections of a synthetic toroidal grayscale point cloud with *N* = 20,000 points; (**a**–**c**) point cloud signal $s(p_{i})$, T-MAHF filter output $r(p_{i})$ magnitude $M_{i}$ and phase $\vartheta_{i}$.

A few remarks are in order regarding the applicability of the method to point clouds with larger numbers of points. In principle, the approach scales naturally to different resolutions, as illustrated in Figure 11 and Figure 12. In these examples, we considered the stereo projections—obtained with the same camera parameters as in [30]—of a synthetic toroidal grayscale point cloud with N=1000 and *N* = 20,000, respectively. For both cases, in addition to the 2D projections, we show the point cloud signal $s(p_{i})$ and the output of the T-MAHF filtering $r(p_{i})$ in terms of its magnitude $M_{i}$ and phase $\vartheta_{i}$, computed from the 2D signal information. In a practical V-SLAM framework, when points are acquired from only two cameras, the 3D point set is restricted to the salient points for which reliable correspondences can be established. Conversely, if the cameras are complemented with Lidar data, the generated point cloud becomes significantly richer.

Finally, we carried out a comparison of the computational complexity of the T-MAHF method with the existing MAHF [25]. The two algorithms share several tasks—such as salient points extraction and matching, and 3D point cloud and mesh computation—but differ in the filtering stage. In T-MAHF a binary adjacency matrix is computed, and filtering is carried out through signal extraction and distance/relative angle computation. In contrast, the MAHF algorithm requires computing the real-valued conformal Laplacian and performing its eigen analysis to calculate the filtering weights as functions of $K_{t}^{\left(G\right)}$ and $\varphi^{\left(G\right)}$. Table 2 reports the computation time of the two methods in milliseconds per point, analyzed on four frames (1,137,981 and 1041 in [30]), and broken down by task. On average, T-MAHF achieves a total computation time reduction of about 40% compared to MAHF. Let us observe that the complexity of the face and edge signal extraction task can increase as far as an increasing number of pixels is considered in the averages in (5) and (6). Still, T-MAHF allows faster point cloud topological signal processing exploiting the potentiality of the T-SLAM framework.

## 5. Conclusions

Topological point cloud processing is gaining momentum, particularly for objects acquired though dedicated multi-camera systems. This paper presents a methodology to build a texture dictionary suited for topological point cloud signal processing. The topology-related dictionary is constructed by extending techniques used in simultaneous localization and mapping (SLAM) algorithms, and adapting them to the point cloud topology description. This approach is useful for various developments where non-Euclidean operators can be applied for processing purposes.The proposed framework, validated through numerical experiments, demonstrated stable heat diffusion consistent with topological distances, reliable approximations of 3D geometric relations from 2D projections, and improved robustness to mesh irregularities. Quantitative analyses further confirmed the effectiveness of TSP-SLAM in enhancing denoising, segmentation, and perceptual quality evaluation tasks, showing clear advantages over graph-based baselines. Future work will focus on extending the framework to more general topological structure, including cell complexes.

## Figures and Tables

**Table 1:** Notation table for variables and parameters.

Notation	Description
$I\left(\tilde{p}\right),I^{\prime }\left({\tilde{p}}^{\prime }\right)$	Stereo images (left and right)
*l*, *b*	Focal length, distance between the left and right cameras
${\tilde{p}}_{i}\;=\;{\left[u_{i},v_{i}\right]}^{T},{\tilde{p}}_{i}^{\prime }\;=\;{\left[u_{i}^{\prime },v_{i}^{\prime }\right]}^{T}$	2D projections of the *i*-th vertex on the left and right images
$p_{i}={\left[p_{ix}\;p_{iy}\;p_{iz}\right]}^{T}$	3D coordinates of the *i*-th point cloud vertex
G	Graph associated to the point cloud
$B_{1}$, $B_{2}$	Graph incidence matrices of first and second order
$L_{0}=B_{1}B_{1}^{T}$	Graph Laplacian matrix of zero order
$L_{1}=B_{1}^{T}B_{1}+B_{2}B_{2}^{T}$	Graph Laplacian matrix of first order
$e_{ij}\;=\;\left\{p_{i},p_{j}\right\}$	Edge incident at the *i*-th, *j*-th vertices
$f_{ijk}\;=\;\left\{p_{i},p_{j},p_{k}\right\}$	Face incident at the *i*-th, *j*-th and *k*-th vertices
${\tilde{e}}_{ij}\;=\;\left\{{\tilde{p}}_{i},{\tilde{p}}_{j}\right\}$	2D projection (segment) of the *i*-*j* edge
${\tilde{f}}_{ijk}\;=\;\left\{{\tilde{p}}_{i},{\tilde{p}}_{j},{\tilde{p}}_{k}\right\}$	2D projection (triangle) of the *i*-*j*-*k* face
$s(p_{i})$	Signal at *i*-th point cloud vertex
$s(e_{j})$	Signal at *j*-th edge
$s(f_{k})$	Signal at *k*-th face

**Table 2:** Computational complexity comparison: computation time (ms per point) for frames 1, 137, 981, and 1041 in [30], measured on an Intel(R) Core(TM) i7-1065G7 CPU @ 1.30 GHz, 1498 MHz, 4 cores, 8 logical processors. On average, the total computation time is reduced by about 40%.

T-MAHF (1.4173 ms per point in average)	
$A\in {\left\{0,1\right\}}^{N\times N}$	[ 0.0494 0.0687 0.0929 0.0494]
$s(f_{i})$	[0.5064 0.1605 0.2718 0.2469]
$s(e_{i})$	[ 0.3064 0.2694 0.4288 0.3350]
Filter weights and computation	[1.4186 0.4354 0.5376 0.4919]
MAHF (2.3894 ms per point in average)	
$L\in R^{N\times N}$	[ 0.4923 0.5932 1.2465 0.5350]
Filter weights ($K_{t}^{\left(G\right)}$)	[ 2.7122 0.8136 1.1894 0.8219]
Filter weights ($\varphi^{\left(G\right)}$	[0.5410 0.1007 0.3300 0.1000]
Filter computation	[ 0.0269 0.0156 0.0247 0.0144]

## Data Availability

The data used in this study are available in the public domain UTIAS Long-Term Localization and Mapping Dataset at http://asrl.utias.utoronto.ca/datasets/2020-vtr-dataset/, accessed on 22 September 2025.
